# Immediate Outcome Indicators in Perioperative Care: A Controlled Intervention Study on Quality Improvement in Hospitals in Tanzania

**DOI:** 10.1371/journal.pone.0065428

**Published:** 2013-06-12

**Authors:** Goetz Bosse, Ferdinand Mtatifikolo, Wiltrud Abels, Christian Strosing, Jan-Philipp Breuer, Claudia Spies

**Affiliations:** 1 Department of Anaesthesiology and Intensive Care Medicine, Campus Charité Mitte and Campus Virchow-Klinikum, Charité – University Medicine Berlin, Berlin, Germany; 2 Bombo Regional Hospital, Tanga, Tanzania; 3 Department of Surgery, Havellandkliniken Klinikum Rathenow, Rathenow, Germany; RAND Corporation, United States of America

## Abstract

**Introduction:**

Outcome assessment is the standard for evaluating the quality of health services worldwide. In this study, outcome has been divided into immediate and final outcome. Aim was to compare an intervention hospital with a Continuous Quality Improvement approach to a control group using benchmark assessments of immediate outcome indicators in surgical care. Results were compared to final outcome indicators.

**Method:**

Surgical care quality in six hospitals in Tanzania was assessed from 2006–2011, using the Hospital Performance Assessment Tool. Independent observers assessed structural, process and outcome quality using checklists based on evidence-based guidelines. The number of surgical key procedures over the benchmark of 80% was compared between the intervention hospital and the control group. Results were compared to Case Fatality Rates.

**Results:**

In the intervention hospital, in 2006, two of nine key procedures reached the benchmark, one in 2009, and four in 2011. In the control group, one of nine key procedures reached the benchmark in 2006, one in 2009, and none in 2011. Case Fatality Rate for all in-patients in the intervention hospital was 5.5% (n = 12,530) in 2006, 3.5% (n = 21,114) in 2009 and 4.6% (n = 18,840) in 2011. In the control group it was 3.1% (n = 17,827) in 2006, 4.2% (n = 13,632) in 2009 and 3.8% (n = 17,059) in 2011.

**Discussion:**

Results demonstrated that quality assurance improved performance levels in both groups. After the introduction of Continuous Quality Improvement, performance levels improved further in the intervention hospital while quality in the district hospital did not. Immediate outcome indicators appeared to be a better steering tool for quality improvement compared to final outcome indicators. Immediate outcome indicators revealed a need for improvement in pre- and postoperative care.

**Conclusion:**

Quality assurance programs based on immediate outcome indicators can be effective if embedded in Continuous Quality Improvement. Nevertheless, final outcome indicators cannot be neglected.

## Introduction

Quality improvement schemes in health care, based on the monitoring of patient outcome such as morbidity and mortality, have gathered increasing attention worldwide. Improved outcome indicators are the ultimate goal of improving the quality of care in health services [1].

The usefulness, however, of final patient outcome indicators such as the Case Fatality Rate as quality indicators for developing countries has been questioned [2]. The fact that outcome indicators are of great intrinsic interest and include all aspects of care has its downside in that these indicators are multi-factorial in nature and influenced by various factors inside and outside the health institutions [3]. If one focuses on improving the hospital-dependent quality of care, numerous confounding factors have to be taken into account and causal attribution can be hard to establish. Furthermore, final outcome indicators are long-term figures, take time to be calculated and are prone to poor documentation, especially in developing countries. The question has been raised if final outcome measures are always appropriate to assess quality [4].

Due to those limitations, there is a need to develop a better understanding of the nature of outcome measures. Hence, it is important to shed more light on the relationship of process and outcome. In 1980, Donabedian already stated that the result of any process in health care might already be a criterion of outcome [1]. According to his view, outcome might begin immediately where an individual process ends and has produced a measurable result. In 2009, a more detailed concept, the logic model, described this connection between process and outcome as a logic chain. In their publication on the logic model in primary care, Watson et al. introduced the terms “immediate outcome” for results that are directly attributable to single processes in health care; and “final outcome” for the long-term objectives of health care services for the population or the health care system [5]. Thus, the end of any process in healthcare is the beginning of outcome and if the standards for single processes are based on evidence-based guidelines outcome is most likely to improve. The authors of this paper will use the terms “immediate outcome” and “final patient outcome” to distinguish between a) results of individual processes that are measured against target standards following evidence-based guidelines and b) population-based long-term results. As an example, the immediate outcome of monitoring a patient after major surgery represents the level of adherence to what is known to be best practice. It also reflects the level to which complications are detected and the patient is most likely to survive. The indicator is available immediately after the procedure has ended. All postoperative monitoring procedures influence the Case Fatality Rate as a final patient outcome indicator of surgical care. Typically, final outcome indicators have to be calculated retrospectively.

### Care Quality Assessment

In 2005, a Hospital Performance Assessment Tool has been introduced in all governmental hospitals in Tanga Region, Tanzania, to systematically review the three categories of quality of care defined by Donabedian (structure, process and outcome) [6], [7]. Both quantitative and qualitative data have been assessed annually and today the assessments are part of the standard quality assurance program for all hospitals in the region.

Tanga Region is one of 26 regions in Tanzania and has an estimated population of 1.7 million. There were no major political, economical or environmental changes during the study period. The last major health sector reform including all regions in Tanzania introduced private fees for health services in governmental hospitals and took place in 1998. Since then, no major reforms have changed the structural or organizational framework of the health care institutions.

The Gross Domestic Product of the Region as a percentage of the National Gross Domestic Product has risen from 4.2% in 2000 to 5.6% in 2006. Up to now, the economy has grown continuously and the region belongs to the wealthier parts of the country.

There are six governmental hospitals providing third level care in the region, one referral hospital and five district hospitals. All hospitals have been assessed annually from 2006 on. All of the hospitals provide surgical care with minor and major surgery. Surgical care is an integral part of health care throughout the world, with an estimated 234 million operations performed annually [8]. Surgical complications, most of which take place in the pre- and postoperative phase, are common and mostly preventable, including in developing countries [9]. A growing body of evidence actually links better performance quality in surgery to improved outcome [9], [10]. Thus, the quality of surgical perioperative care from admission to discharge is crucial and emphasis on improving and maintaining the quality of care is vital.

In our study, immediate outcome indicators of the health services in the surgical department were assessed in comparison to a target standard that has been jointly developed by Tanzanian and German partners incorporating international and national guidelines of best practice. A benchmark for good quality for each key procedure was purposely agreed to be 80% of the target standard, knowing that in other contexts 70% was suggested to be sufficient [11].

### Underlying Quality Improvement Approach

In the referral hospital, the assessment was part of a Continuous Quality Improvement Approach to improve the quality of care according to the “Plan-Do-Check-Act” strategy as described first by Deming [12]. This implied that after the annual assessment of care, quality areas in need of improvement and areas of strength were identified. Consecutively, a Hospital Quality Team performed supervised quality circles in which individual interventions were defined to specifically address areas in need of improvement. After one year, another quality assessment was conducted in order to evaluate the changes in care quality thereby initiating another cycle of assessment and intervention.

### Aim of the Study

The aim of the study was to compare the levels of care quality in the intervention hospital to those of a control group of hospitals, where no Continuous Quality Improvement Approach had been installed. The comparison was conducted over five years with benchmark assessments of immediate outcome indicators in comparison to final outcome indicators. Thus, the effect of the additional quality improvement approach together with quality assurance in comparison to quality assurance alone was investigated. At the same time, the usefulness of immediate outcome indicators in addition to final patient outcome was evaluated.

## Methods

In this controlled study data authorization and ethical committee approval was obtained from Tanzanian authorities as an amendment to ethical clearance reference number NIMR/HQ/P.12/Vol.VIII/27.

### The Hospital Performance Assessment Tool

In 2005, a Hospital Performance Assessment Tool has been introduced in all governmental hospitals in Tanga Region, north-eastern Tanzania [6]. The tool systematically reviews the quality of care in Tanzanian hospitals through observation of processes and patient file review as well as interviews with health care workers and patients. It is based on Donabedian’s structure, process and outcome model [1].

In a consensus process with the Tanzanian partners, checklists were jointly developed on the basis of national and international evidence-based guidelines. They were structured in key procedures for all clinical, supportive and non-clinical departments of the hospitals that were termed “focal points” in the methodology of the tool. Focal points include the clinical departments of Maternity, Surgery, Pediatrics and Medicine; the diagnostic departments of Radiology, Laboratory, Blood Bank and Pharmacy; the non-clinical focal points Management, Maintenance, Waste and Hygiene, Water and Power. Also structural quality was assessed with a checklist in all focal points respectively.

Checklists comprised of 5–15 single items for process quality and up to 38 items for structural quality that were assessed on a Likert scale from 0 (0%) to 2 (100%). A “How to do it” column was designed in the checklist with cut-off levels for each individual item. In assessing care quality this way, not only performance was compared to a standard, but also the standard was the guideline for best clinical practice. Target standard was a 2 on the Likert scale corresponding with 100% expected performance level. Thus, the actual performance level for every key procedure was assessed as the sum of the individual item scores divided through the target standard of 2 (100%) per item [6]. It was expressed as percentage of the expected performance level.

Thus, every key procedure had an immediate outcome indicator, reflecting the adherence to checklists based on evidence-based standards.

**Figure 1:**
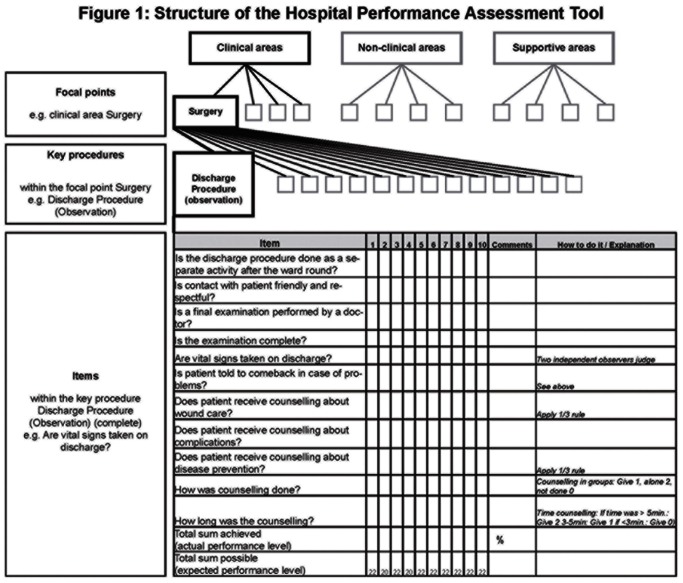
Structure of the Hospital Performance Assessment Tool. There are twelve focal points (maternity, surgery, pediatrics, medicine, laboratory, pharmacy, radiology, blood bank, management, maintenance, waste and hygiene, water and power). Key procedures consist of individual items that are structured in form of checklists. The assessment is conducted with the checklist of the items. The checklist with all items for the key procedure “discharge (observation)” in the clinical focal point surgery is given.

For an overview how the tool has been structured in focal points, clinical key procedures and individual items see Figure 1. Figure 1 also displays the checklist for the key procedure discharge (observation) in the focal point Surgery as an example. In total, checklists comprised of 1162 process items structured in clinical key procedures. All departments of the respective hospital were observed throughout the entire process of care, from admission until discharge. Observers also conducted interviews with both patients and staff. Only quantitative data was included in this study.

### Assessment in this Study

For this study, the quality assessments with the Hospital Performance Assessment Tool as described above were performed by two independent observers from the group of health workers in the respective hospital. To minimize observer bias, observers collected their results individually and compared them afterwards. In case of disagreement procedures were reassessed. Furthermore, they were supervised by members of the Regional Health Management Team. One assessment took an average of 3 working days. Assessment data from 2006, 2009 and 2011 were included in this study. Changes in immediate outcome indicators of individual key procedures in the surgical department were analyzed and compared between the intervention hospital and the control group over the study period. They were also compared to the surgical Case Fatality Rates. However, there were no reliable Case Fatality Rates for all surgical departments in the control group, thus results were also compared to the Case Fatality Rates of all in-patients in both groups. Those final outcome indicators were taken from the annual regional reports and the hospital reports of the intervention hospital.

The benchmark for good quality was agreed to be 80%, knowing that in other contexts 70% was suggested to be sufficient [11]. Benchmarking is a way of systematically comparing actual performance quality in health services to a pre- and locally defined standard [13], as it is done in the assessment tool. Since benchmark assessments of immediate outcome indicators are originating in their processes, they are likely to reflect precisely the quality of care in the hospital and its changes.

### Underlying Quality Improvement Approach

The underlying quality improvement concept follows a Continuous Quality Improvement Approach, which has been suggested to be especially useful in developing countries [14]. Hence, the Hospital Performance Assessment Tool is one step of a circulatory process in the sense of the “plan-do-study-act”-circle, also known as Deming cycle [12]. After the assessment of care quality, results were presented and discussed with the Hospital Quality Team, a group appointed by the hospital management to entirely concentrate on quality issues of the hospital. The team consisted of 12 members, selected from different cadres of health workers from all departments and the hospital management. After presenting the assessment results, the Hospital Quality Team performed a supervised quality circle in which interventions were defined and documented in an Action Plan to specifically address areas in need of improvement. Responsibility for single interventions was assigned to individual health workers respectively, and a time frame was agreed on and documented. The Hospital Quality Team appointed a supervisor to monitor the implementation of the interventions. This person reported the status of implementation back to the Hospital Quality Team in their monthly Hospital Quality Team meeting. After one year, another quality assessment was conducted in order to evaluate changes and initiate another cycle.

### Selection of Control Group

The quality improvement approach as described above has been installed so far only in the referral hospital. Thus, the referral hospital is the intervention hospital in this study. It represents the standard governmental reference hospital in the Tanzanian health system. In 2006, it was the biggest hospital in the region with 395 beds and three individual wards in the surgical department. There were 349 health workers including 18 Medical Officers and Assistant Medical Officers. The Assistant Medical Officer program was created more than 40 years ago to compensate for the low output of medical universities in Tanzania. Requirement for becoming an Assistant Medical Officer is outstanding performance as a nurse for a time period of at least three years and an additional full-time 2-year intensive education program. Assistant Medical Officers act as fully certified doctors in the Tanzanian health system.

There was no quality improvement approach in the five district hospitals, but only annual assessments of care quality under the supervision of the Regional Health Management Team. One of the hospitals was not included into the control group, because it was a former missionary hospital and thus not fully governmental in terms of structural quality and process organisation. In another hospital there had been an attempt to introduce an action plan in 2009 that was not followed up afterwards due to the lack of a Quality Team. The other three hospitals were pooled in one control group on the basis that they are all governmental, and furthermore similar in terms of structural quality, process organization and bed capacity. In 2006, in the three hospitals together, there were 298 beds and 657 health workers including 20 Medical Officers and Assistant Medical Officers. There was one surgical ward in each hospital of the control group respectively.

In summary, the three hospitals in the control group represented the standard third level care institution in the Tanzanian health system providing regular health care services. They were pooled together in one control group to make it comparable to the intervention hospital in terms of bed capacity, number of medical personnel, and number of surgical wards. A detailed list of hospital characteristics and developments over the study period is given in the results section (Table 1).

**Table 1:** Hospital characteristics and their development over the study period.

Hospital characteristics 2006, 2009, 2011						
	Intervention hospital			Control group		
Year →	2006	2009	2011	2006	2009	2011
Indicator ↓						
**Number of beds**	395	392	392	298	324	335
**Number of health workers**	349	370	382	657	376	418
**Number of Medical Officers and Assistant Medical Officers**	18	27	27	20	25	37
**Number of admissions per year**	15500	21114	18840	17827	13632	17059
**Case Fatality Rate for all in-patients**	5.5%	3.5%	4.6%	3.1%	4.2%	3.8%
**Number of admissions in surgery**	2736	4035	3654	n/a	n/a	n/a
**Number of surgical wards**	3	3	3	3	3	3
**Number of major surgery performed** 1	254	427	409	756	904	735
**Emergency procedures in surgery**	38.4%	54.7%	56.0%	n/a	n/a	n/a
**Case Fatality Rate for all surgical in-patients**	n/a	4.3%	2.9%	n/a	n/a	n/a
**Average length of stay**	5.8	5.2	5.3	n/a	n/a	n/a
**Average bed occupancy rate**	62.3%	72.0%	69.0%	n/a	n/a	n/a

1 (without cesarean section and ophthalmology).

Characteristics of the intervention hospital and the control group hospitals in comparison over the study period. “n/a” indicates not available.

### Statistical Analysis

Immediate outcome indicators were expressed and compared as proportions [%]. Considering all indicators as equally powerful, the significance of differences of the actual performance levels in 2006, 2009 and 2011 was tested with chi-square-test. All tests were carried out in exact versions. A two-tailed p<0.05 was considered statistically significant. Analysis was done with IBM® SPSS® Statistics, Version 20, © IBM Corporation, Armonk, NY, USA.

## Results

In 2006, the referral hospital was the biggest hospital in the region with 395 available beds and 349 health workers on a permanent basis. The district hospitals in the control group of this study had a total of 298 beds and 657 health workers. There was one surgical ward in each hospital of the control group respectively. For the development of hospital characteristics over the study period see Results Table 1.

### Immediate Outcome Indicators

**Figure 2:**
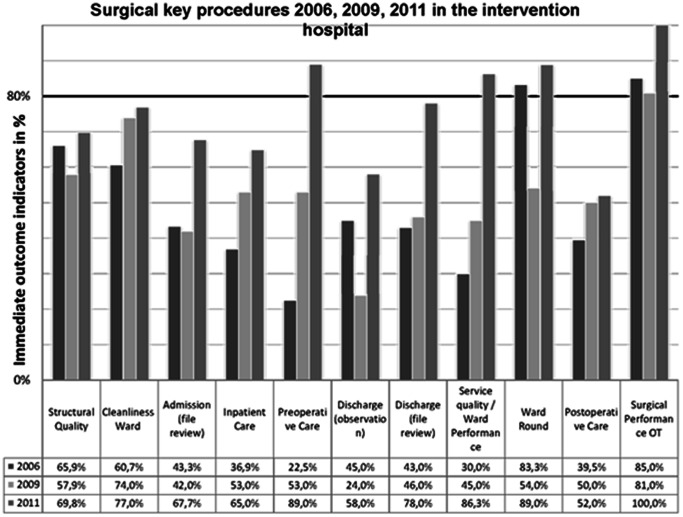
Surgical key procedures over 2006, 2009, 2011 in the intervention hospital. The black line indicates the benchmark of 80%. One key procedure (ward round, surgical performance) score over benchmark in 2006, one (surgical performance) in 2009 and in 2011. There are four key procedures (preoperative care; ward performance; ward round; surgical performance) with an immediate outcome indicator of more than 80%.

In the intervention hospital, six key procedures improved significantly towards 2011, namely admission (p<0,001), inpatient care (p<0,001), preoperative care (p<0,001), discharge in files review (p<0,001), ward performance (p<0,001) and surgical performance (p<0,001). Two out of nine key procedures reached the benchmark of 80% (ward round; surgical performance) in 2006, whereas this was one out of eleven in 2009 (surgical performance), and four out of nine in 2011 (preoperative care; ward performance; ward round; surgical performance). Structural quality in the surgical department was 65.9% in 2006, 57.9% in 2009 and 69.8% in 2011. For all immediate indicators in the intervention hospital see Table 2 and Figure 2.

**Table 2:** Surgical key procedures over 2006, 2009 and 2011 in the intervention hospital.

Intervention Hospital – surgical key procedures					
Year →	2006 [%]	2009 [%]	2011 [%]	p-value 2006–2009	p-value2006–2011
↓ Key procedures					
**Structural Quality:**					
**Structure surgical wards**	65.9 (n = 1)	57.9 (n = 1)	69.8 (n = 1)	0.31	0.65
**Process Quality:**					
**Admission**	43.3 n = 18	42.0 n = 10	67.5*↑ n = 20	0.99	<0.001
**Inpatient care**	36.9 n = 18	53.0*↑ n = 10	61.6*↑ n = 11	0.03	<0.001
**Preoperative care**	22.5 n = 10	53.0* **↑** n = 10	**84.8** * **↑** n = 20	<0.001	<0.001
**Discharge (observation)**	45.0 n = 10	24.0*↓ n = 10	55.7 n = 20	0.01	0.09
**Discharge (file review)**	43.0 n = 10	46.0 n = 10	74.3*↑ n = 18	0.78	<0.001
**Service quality/Ward performance**	30.0 n = 1	45.0* n = 1	**86.4** * **↑** n = 3	0.04	<0.001
**Ward round**	**83.3 **n = 1	54.0*↓ n = 1	**88.9 **n = 3	<0.001	0.31
**Postoperative care**	39.5 n = 10	50.0 n = 10	48.3 n = 10	0.16	0.32
**Operating theatre surgical performance**	**85.0 **n = 10	**80.0 **n = 5	**100** * **↑**n = 1	0.99	<0.001
**Mean performance level of surgical key procedures**	47.6	49.7	76.1*↑	0.89	<0.001

Immediate outcome indicators in surgical care in the intervention hospital 2006, 2009, 2011.

* indicates significant change,

↑indicates improvement,

↓indicates decline.

**Figure 3:**
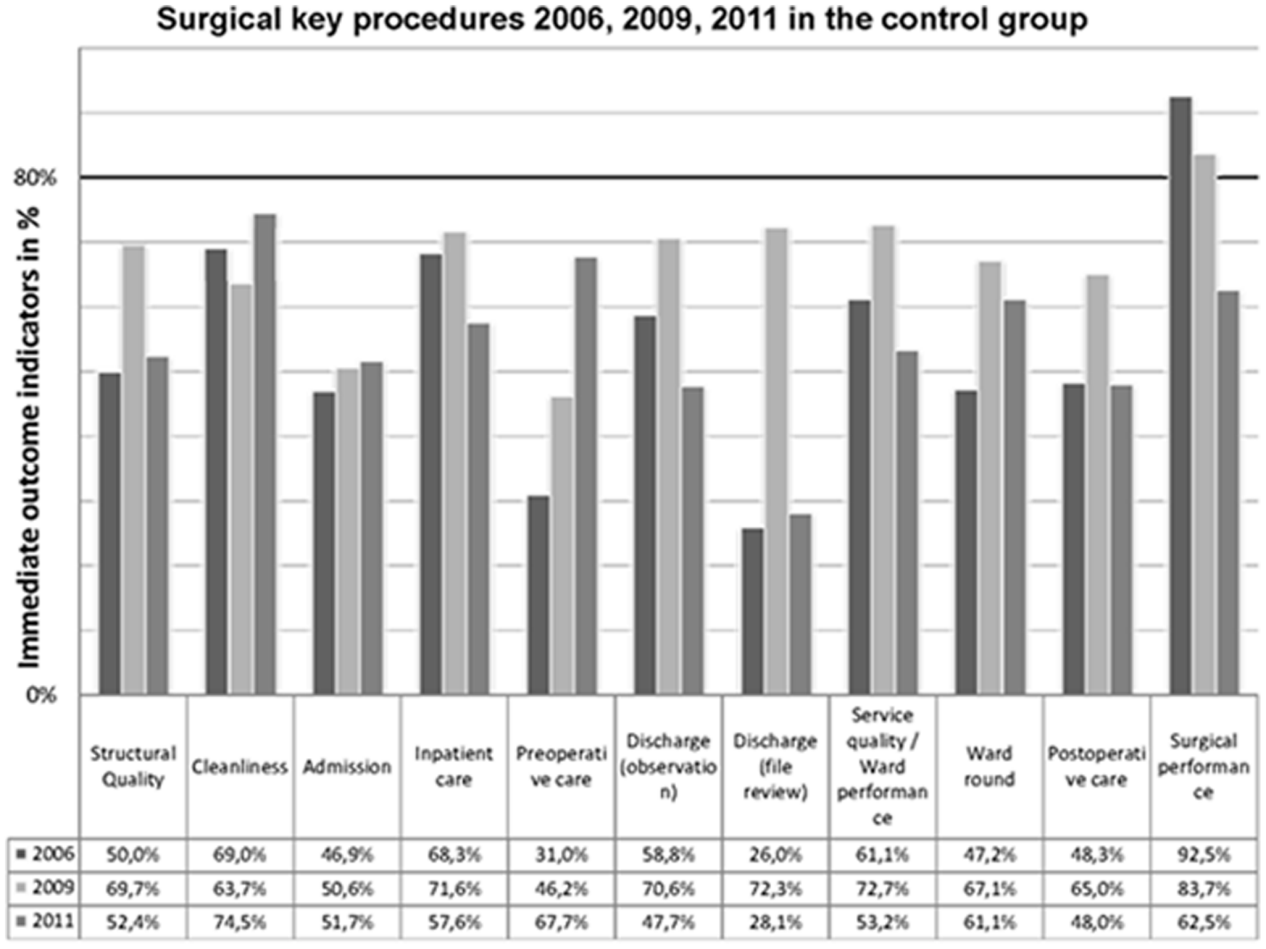
Surgical key procedures over 2006, 2009, 2011 in the control group. The black line indicates the benchmark of 80%. One key procedure (surgical performance) scores over benchmark in 2006, one (surgical performance) and in 2011 there is no key procedures with an immediate outcome indicator of more than 80%. Five procedures (discharge (observation); inpatient care; ward performance; postoperative care; surgical performance) have lower performance levels in 2011 than in 2006.

In the control group, one key procedure improved significantly towards 2011, namely preoperative care (p<0,001). One out of nine key procedures reached the benchmark of 80% (surgical performance) in 2006, the same indicator reached the benchmark in 2009 (surgical performance), and none of the key procedures reached the benchmark in 2011. Structural quality in the surgical department was 50.0% in 2006, 69.7% in 2009 and 53.1% in 2011. For all immediate indicators in the control group see Table 3 and Figure 3.

**Table 3:** Surgical key procedures over 2006, 2009 and 2011 in the control group.

Control group – surgical key procedures					
Year →	2006 [%]	2009 [%]	2011 [%]	p value 2006–2009	p value 2006–2011
↓ Key procedure					
**Structural Quality:**					
**Structure surgical wards**	50.0 n = 3	69.7 n = 3	53.1 n = 3	0.01	0.78
**Process Quality:**					
**Admission files**	46.9 n = 30	50.6 n = 30	51.7 n = 10	0.67	0.57
**Inpatient care**	68.3 n = 3	71.6 n = 27	57.6 n = 10	0.65	0.19
**Preoperative care**	31.0 n = 22	46.2 * **↑** n = 26	67.7* **↑** n = 6	0.03	<0.001
**Discharge (observation)**	58.8 n = 4	70.6 n = 30	47.7 n = 6	0.1	0.16
**Discharge (file review)**	26.0 n = 24	72.3* **↑** n = 28	28.1 n = 8	<0.001	0.87
**Service quality/Ward performance**	61.1 n = 3	72.7 n = 3	53.2 n = 3	0.22	0.32
**Ward round**	47.2 n = 3	67.1* **↑** n = 3	61.1 n = 3	0.01	0.07
**Postoperative care**	48.3 n = 12	65.0* n = 27	48.0 n = 7	0.02	0.99
**Operating theatre surgical performance**	**92.5 **n = 5	**83.7 **n = 13	62.5* **↓** n = 3	0.35	<0.001
**Mean performance level of surgical key procedures**	53.3	66.6	53.1	0.06	0,99

Immediate outcome indicators in surgical care in the control group 2006, 2009, 2011.

* indicates significant change,

↑indicates improvement,

↓indicates decline.

For an overall trend, mean performance levels were calculated. The mean performance levels for all surgical key procedures in the intervention hospital were 47.6% in 2006, 49.7% in 2009 and 76.1% (p<0,001) in 2011. The mean performance levels for all surgical key procedures in the control group were 53.3% in 2006, 66.6% in 2009 and 53.1% (p = 0.99) in 2011 (see Table 2 and Table 3).

In 2011, the three surgical wards in the intervention hospital were assessed individually to analyse performance quality under the same structural conditions. The mean performance level on ward A1 was 65.6%, on ward A2 71.4% and on ward 7 80.4%. Structural quality in 2011 was 75.0% on A1, 71.1% on A2 and 63.2% on ward 7. For details, see Table 4.

**Table 4:** Surgical key procedures in 2011 in the individual surgical wards of the intervention hospital.

Intervention hospital 2011– individual surgical wards			
Surgical wards →	A1 [%]	A2 [%]	7 [%]
↓ Key procedures			
**Structural Quality:**			
**Structure surgical wards**	75,0 n = 1	71,1 n = 1	63,2 n = 1
**Process Quality:**			
**Admission files**	48,0 n = 5	67,0 n = 10	**88,0 **n = 5
**Inpatient care**	47,0 n = 5	78,0 n = 3	70,0 n = 3
**Preoperative care**	**100,0 **n = 5	**81,0 **n = 5	**86,0 **n = 10
**Discharge observed**	64,0 n = 5	49,0 n = 10	61,0 n = 5
**Discharge files**	73,0 n = 5	67,0 n = 9	**94,0 **n = 4
**Service quality/** **Ward performance**	73,0 n = 1	**91,0 **n = 1	**95,0 **n = 1
**Ward round**	**83,0 **n = 1	**92,0 **n = 1	**92,0 **n = 1
**Postoperative care**	37,0 n = 5	62,0 n = 3	57,0 n = 2
**Mean performance level of surgical** **key procedures**	65, 6	73,4	**80,4**

Individual immediate outcome indicators in the three surgical wards of the intervention hospital in 2011.

### Final Outcome Indicators

The surgical Case Fatality Rate in the intervention hospital was not available in 2006, 4.3% in 2009 (n = 4,035) and 2.9% in 2011 (n = 3,654). There was no specific Case Fatality Rate available for the surgical departments of the control group.

The Case Fatality Rate for all in-patients in the intervention hospital was 5.5% (n = 12,530) in 2006, 3.5% (n = 21,114) in 2009 and 4.6% (n = 18,840) in 2011.

The Case Fatality Rate for all in-patients in the control group hospitals was 3.1% (n = 17,826) in 2006, 4.2% (n = 13,632) in 2009 and 3.8% (n = 17,059) in 2011.

## Discussion

The most important findings were:

- With the introduction of quality assessment, performance levels improved in both groups. After the introduction of a Continuous Quality Improvement scheme in the intervention hospital, performance levels improved further and in more areas while quality in the district hospital did not.
- In comparison to final outcome indicators, immediate outcome indicators appeared to be more consistent, precise and a good tool to steer quality improvement.
- Immediate outcome indicators demonstrated precisely areas of improvement and those of concern. For the actual surgical performance they showed sufficient quality in both the intervention hospital as well as the control group. In pre- and postoperative care they demonstrated a need for improvement.
- Wards within the same department performed differently under the same structural conditions.

### Quality Assurance versus Quality Improvement

With the introduction of quality assessment, performance levels did improve after 2006 in the intervention hospital and in the control group. However, after introduction of a Continuous Quality Improvement scheme in the intervention hospital, performance levels improved further and in more areas in the intervention hospital, while quality in the control group did not improve further. In fact, immediate outcome indicators worsened in the control group towards 2011 in both process and structural quality. Several studies on the quality of care in developing countries call for improvement of structural quality [15]–[17]. Although this cannot be neglected, the authors agree with the view of Reerink et al, who already stated in 1996 that the improvement of care quality is not tantamount to improving structural quality. Instead, the authors suggest, that focusing on process improvement is the most promising avenue for improving quality of care in developing countries [18].

Reerink et al. also suggested improving the quality of care through quality assurance programs [18]. Our results from the control group indicate that the mere assessment of care quality does indeed improve performance quality to a certain level but not beyond. In the intervention hospital, after 2009, the hospital management decided to act upon results with specific activities tailored to areas in need of improvement, and immediate outcome indicators improved further. Thus, we agree with the view of Guinane et al., that coordinated actions have to be implemented along the lines of the Deming cycle. According to them, if quality assurance and quality improvement are integrated in this way, processes and outcomes can be improved [19].

Agyepong et al. state that it can take up to five years to build up organization-wide confidence in a Continuous Quality Improvement approach, but strongly emphasize the point that this approach is especially important in the context of a developing country where multi-factorial and deep-rooted problems are unlikely to respond to any overnight solution [14].

In summary, there is little evidence that quality assurance or quality management improves final outcome [20], our study contributes to that notion. On the basis of our results we suggest that quality assurance can be effective, if given enough time and embedded in a continuous improvement program.

### Immediate Outcome Indicators versus Final Outcome

The improvement of immediate outcome indicators in the intervention hospital was reflected in a decrease of the surgical Case Fatality Rate as well as in the Case Fatality Rate for all in-patients, while structural quality did hardly improve. In the control group, immediate outcome indicators did not improve towards 2011 but rather declined again to the level of 2006. There was a significant peak in 2009 that may be attributed to the introduction of quality assurance. There was no surgical Case Fatality Rate available for direct comparison with the intervention hospital, but the Case Fatality Rate for all in-patients did slightly decline. Immediate outcome indicators did not improve to the extent of the intervention hospital. Considering the complex nature of final outcome indicators and poor quality of documentation in many developing countries one has to be cautious to draw conclusions from these findings too quickly. However, immediate outcome assessments suggest that the quality improvement program did indeed improve the quality of care in the intervention hospital significantly. We propose that immediate outcome indicators have several advantages if one is focusing on the hospital-based quality of care. Lilford et al. suggested that direct measures of performance, such as immediate outcome indicators, immediately reflect improvements in performance quality [21]. Thus, they are quick to assess and specific as they reflect the processes they monitor. Moreover if compared to final outcome indicators, they are more sensitive to changes in quality and need less numbers of cases to specifically reflect upon areas of strengths and weaknesses in a hospital [3]. Finally, validated process measures provide an important additional element to quality improvement efforts, as they illuminate exactly which provider actions could be changed to improve patient outcomes [22]. This characteristic is especially valuable in the quality improvement approach used in this study.

Final outcome indicators, on the other hand, are subject to external influences and depend on large numbers that have to be collected and analyzed. We suggest that if final outcome indicators are to guide the daily performances, too many patients are hindered to receive good quality of care as long as they are not yet available. Instead of waiting for final outcome indicators to be calculated it might be more helpful to use immediate outcome indicators as a target for the quality of individual processes and improve the performances along these results [3].

On the basis of our findings we conclude, that the positive effects of a quality improvement scheme can be evaluated with immediate outcome indicators. Moreover, the use of process-based immediate outcome indicators is a better steering tool towards improved performance quality in the hospital than final outcome indicators. Nevertheless, final outcome indicators reflect all aspects of care and are of great intrinsic interest [3] and can therefore not be neglected as the desired endpoints of health care [23], [24].

### Immediate Outcome Indicators: Surgical Performance and Pre- and Postoperative Care

In this study, the actual performance of surgical procedures in the operating theatre was found to be satisfactory with the exception of the 2011 data of the control group. However, the quality of postoperative care did reach its benchmark in the whole study period neither in the intervention hospital nor in the control group. The quality of preoperative care reached its benchmark only in 2011 in the intervention hospital. Results suggest that pre- and postoperative care of patients, including monitoring and nursing care need to be improved. Several authors support these findings. In a study on paediatric hospital care in seven different developing countries, Nolan et al. found that 76% of the patients received poor monitoring [25] and in another study only in 4% of the inpatient children heart rate was documented [26]. Experiences from Kenya suggest that these problems do not seem to exist exclusively in pediatrics [27]. Findings from South Africa show poor monitoring in primary health care for cases of HIV, tuberculosis and sexually transmitted diseases [28].

### Quality Differences between Wards under the Same Conditions

In 2011, the surgical wards in the intervention hospital were assessed individually. One of the wards scored markedly better than the other two under notably worse structural conditions. These differences strengthen the point that improving performance in health care services does not depend upon structural quality alone. Thus, one hypothesis is that with the resources present there is a chance to improve the quality of care [29]. While a minimum level of inputs is necessary to maintain a meaningful level of care [29], Reerink et al. described an ‘inappropriate focus on inputs’ when trying to improve quality of care in developing countries [18]. Further studies are needed to investigate what level of structural quality is necessary to support processes quality.

## Limitations

### Case Mix

In this study, developments in care quality in hospitals with and without quality improvement schemes have been assessed. There are some methodological concerns. As the reference institution, the regional hospital is likely to receive more severe cases than the control group hospitals. It has been stated that comparisons of performance in healthcare can only be done if it is taken into account whether the measures being compared derive from similar patient groups. Usually, scoring systems are used in order to describe the risk level of individual patients including age, severity of disease, co-morbidities and past medical history [30]. In this study, though, specific adjustment for case mix has not been applied due to the routine nature of data collection and the design of the tool. It has been stated that when process measures are used to drive quality improvement, the problem of case mix largely ends, where appropriate processes of care for specific patient groups have been defined [30], as in the conceptual design of this tool. We are comparing actual performance to a predefined standard. While high risk patients depend on different standards than non-risk patients, performance quality for both groups can be assessed against their respective standard.

Mant el al. raised the question, under which circumstances it is worth going through the effort and the expenses of setting up an outcome monitoring system covering adequate numbers of patients with consistent methods of definition and including sufficient case-mix data for risk-adjustment, when process measures are easier to interpret and less costly [3]. It would be misleading to assume that final outcome indicators are to be neglected or that case-mix is a concept only applicable in developed countries, and thus the conclusions drawn from this study are limited. However, the authors suggest that a developing country might be likely to be a surrounding where the advantages of continuing quality assessment of immediate outcome measures outweigh methodological concerns regarding the case mix.

### Case Fatality Rate and Documentation

Quality of documentation in developing countries has been reported to be not always trustworthy [18]. In our study we found that the Case Fatality Rate seems an unreliable measure due to the fact that it depends largely on good documentation. However, there are no other final outcome indicators available. We believe, that besides the necessary improvement of documentation skills in the hospital, it is of utmost importance to countercheck documentation by observation and interviews, as it is done in the Hospital Performance Assessment Tool.

### Structural and Process Quality

In the intervention hospital improvement of process quality appeared to be independent from structural quality. This is in line with the findings from Reerink and Sauerborn that the improvement of process quality is not tantamount to improving inputs [18].

In the control group structural quality and process quality seemed to draw a parallel. Whether this is be due to the relative small amount of data or the absence of a quality improvement program or other reasons will have to be subject of further research.

### Conclusions

Benchmark assessments of immediate outcome indicators originate in the processes they reflect. They are faster than final outcome indicators and more specific.

Through immediate outcome indicators we found that deficiencies in surgery can be immediately linked to single procedures, e.g. the quality of patient care in the pre- and postoperative phase.

On the basis of our results, we believe that a quality assurance program based on immediate outcome indicators is a better steering tool to improve quality of care, if embedded in a Continuous Quality Improvement approach. Nevertheless, final outcome indicators are the desired endpoints of healthcare and cannot be neglected.
